# High-Flux Hemodialysis and Levocarnitine in the Treatment of Severe Valproic Acid Intoxication

**DOI:** 10.1155/2013/526469

**Published:** 2013-05-21

**Authors:** V. Temel, Müge Arikan, G. Temel

**Affiliations:** Department of Anesthesia, Karabük State Hospital, Karabük, Turkey

## Abstract

Valproic acid (VPA) intoxication incidence is increasing, because of the use of VPA in psychiatric disorders. The most common finding of VPA intoxication is central nervous system depression which leads to coma and respiratory depression. Pancreatitis, hyperammonemia, metabolic, and bone marrow failure (thrombocytopenia and leukopenia) have also been described. Treatment is mainly supportive. We present the case of an 18-year-old female patient, who made an attempt to autolysis with VPA. Our patient's VPA plasma level was very high (924 **μ**g/mL), confirming that it was a severe intoxication. Our treatment including levocarnitine (50 mg/kg per day for 3 days), and high-flux hemodialysis was performed for four hours. The patient's hemodynamic status and mental function improved in conjunction with the acute reduction in VPA concentrations. Her subsequent hospital course was complicated by transient thrombocytopenia and levocarnitine induced hypophosphatemia. By day 6, the patient's laboratory values had completely normalized, and she was transferred to an inpatient psychiatric facility for continuing therapy.

## 1. Introduction

Valproic acid (VPA) is increasingly used in the treatment of epilepsy and also prescribed for bipolar affective disorders, schizoaffective disorders, schizophrenia, and migraine prophylaxis [1]. 

Valproic acid intoxication with suicide attempt is a relatively common clinical problem that can result in coma, respiratory depression, pancytopenia, hemodynamic instability, and death [2].

The treatment of VPA toxicity is mainly supportive. Anecdotal reports describe the efficacy of naloxone and levocarnitine, but the data are insufficient to make strong conclusions [3]. Various extracorporeal techniques for managing VPA toxicity have been described, but none has prevailed as standard therapy [4]. 

We present a patient in which high-flux hemodialysis and levocarnitine were administered for severe VPA intoxication.

## 2. Case Report

An 18-year-old woman was brought in by ambulance to the Emergency Department (ED) one hour after intentional ingestion of 60 VPA tablets (500 mg VPA, >600 mg/kg). Her medical history included partial symptomatic epilepsy and she was medicated with 500 mg per day VPA since two years. 

On ED arrival, the patient was confused and somnolent. Her Glasgow Coma Scale (GCS) was 8. Vital signs included a temperature of 98.7°F, pulse of 129 beats/minute, respiration of 32 breaths/minute, blood pressure of 80/57 mmHg, and puls oximetry (SpO_2_) of %80 on room air. Pupils were equal at 2 mm and reactive to light. The cardiovascular examination revealed a regular rhythm with notable tachycardia without murmurs. The lungs were clear. The abdomen had positive bowel sounds and was soft, nontender, and with no hepatosplenomegaly. The extremities revealed no clubbing, cyanosis, or edema.

In the ED, she was treated with gastrointestinal decontamination with a single dose of activated charcoal and intravenous fluids resuscitation. Her blood VPA level at admission was 844 *μ*g/mL (therapeutic serum concentrations range from 50 to 125 *μ*g/mL) and ammonia level 325 *μ*g/dL. Other laboratory values upon admission were within normal limits. Electrocardiogram revealed sinus tachycardia with no ischemic changes. Her initial chest X-ray and head CT were unremarkable. During hospitalization she was still confused and lethargic but required neither airway protection nor respiratory support.

She was taken to the intensive care unit for continuous monitoring and due to elevated VPA level (928 *μ*g/mL). Her level of glucose was 64 mg/dL, so 25% dextrose 100 mL was administered. Levocarnitine treatment was started (50 mg/kg per day). Nephrology was consulted for evaluation for hemodialysis. Nephrology's opinion was that she could benefit from dialysis. Urgent high-flux hemodialysis (HD) with FX80 dialyzer (helixone, 1.8 m^2^, Fresenius Medical Care), blood flow rate 350 mL/min, and ultrafiltration rate 450 mL/hr for duration of 4 hours was performed, using right femoral venous access. After dialysis, her VPA levels dropped to 292 *μ*g/mL. The serial VPA levels and laboratory studies were followed. Her VPA levels and other investigations are shown in Table 1. 

**Figure 1:**
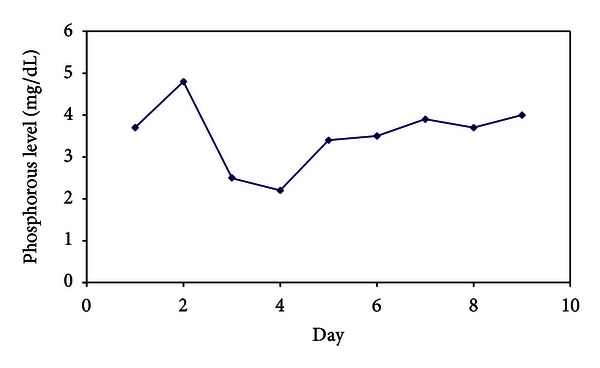
Patient phosphorous level.

**Figure 2:**
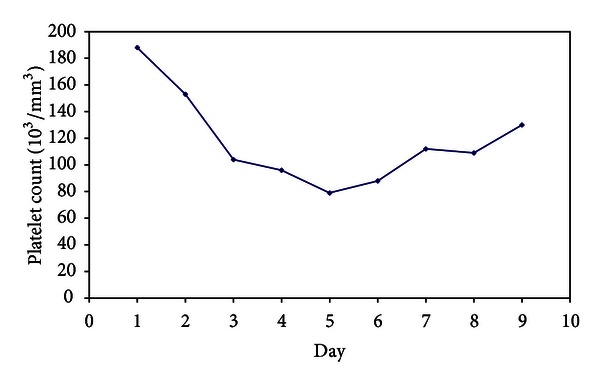
Patient platelet count.

The patient's mental status gradually improved back to baseline over 48 hours, accompanied by decreasing VPA levels (151 *μ*g/mL). Her oral feeding was started on the 3rd day. VPA 500 mg per day was restarted on day 3 of hospitalization. After 3 days of levocarnitine, her phosphorous level fell to 2.2 mg/dL from 3.7 mg/dL before levocarnitine which was discontinued. Her phosphorous increased to 3.4 mg/dL (Figure 1). By day 4, the patient's platelets count decreased to 79 × 10^9^/L from 188 × 10^9^/L and was normalized after 2 days without complications (Figure 2). She was observed closely for any deterioration in consciousness along with VPA levels and laboratory values. By day 6, the patient's laboratory values had completely normalized, and she was transferred to an inpatient psychiatric facility for continuing therapy.

## 3. Discussion

Valproic acid overdose is usually well tolerated. Intoxication usually only results in mild central nervous system (CNS) depression, but serious toxicity and death have been reported [1]. 

Symptoms of VPA intoxication are diverse and are related to VPA plasma concentration. Although total plasma concentrations of less than 450 *μ*g/mL produce limited toxicity, severe intoxications (>850 *μ*g/mL) can induce coma and are ultimately life threatening [1]. 

Central nervous system depression is the most common manifestation of toxicity, ranging in severity from mild drowsiness to profound coma and fatal cerebral oedema [5]. The patients who ingest more than 200 mg/kg VPA and/or have plasma concentrations greater than 180 *μ*g/mL usually develop severe CNS depression [5]. 

In our case, the patient took enteric-coated delayed-release preparation 600 mg/kg and serum levels taken 2 and 4 hours after ingestion were very high (resp., 844 and 925 *μ*g/mL). Her GCS was 8.

Other clinical manifestations of severe VPA intoxication include hypotension, respiratory depression, bone marrow failure (thrombocytopenia and leukopenia), hypothermia or fever, tachycardia, miosis, agitation, hallucinations, tremors, myoclonus, hypoglycaemia, seizures, and hyperammonemia [5]. Bone marrow effects of VPA are well recognized in overdose. Spiller et al. [6] demonstrated that thrombocytopaenia occurred with valproate concentrations >450 *μ*g/mL. Our patient showed hypotension, tachycardia, hypoglycaemia, hyperammonemia, and thrombocytopaenia.

Management of acute VPA intoxication is largely supportive. Patients who present early may benefit from gastrointestinal decontamination with a single dose of activated charcoal. Other interventions may involve blood pressure support with intravenous fluids and vasopressors, as well as correction of electrolyte abnormalities or acid-base disorders (commonly an anion gap metabolic acidosis). Mechanical ventilation may be necessary in patients who require airway protection or who develop cerebral oedema or respiratory depression [6]. 

Levocarnitine may confer potential benefit in VPA-toxicity, especially in the setting of hepatotoxicity, hyperammonemia, or significant CNS depression (50 to 100 mg/kg per day) [7]. A small study of levocarnitine in patients with severe VPA toxicity demonstrated a significant mortality benefit. Levocarnitine's mechanism of action is thought to be related to its ability to decrease elevated ammonia levels, which may contribute to development of coma in VPA toxicity [8]. Levocarnitine has been shown to reduce phosphorus levels. Prohaska et al. [9] presented a case in which levocarnitine was administered for VPA intoxication, and after two days of treatment, phosphorous level decreased. We used levocarnitine in our patient (50 mg/kg per day for 3 days) and hypophosphatemia was occurred.

Patients with significant neurological and cardiovascular effects or with a VPA levels (>850 *μ*g/mL) should be considered for hemodialysis. VPA is highly protein bound, and saturation of available binding sites usually occurs when blood levels reach 90–100 *μ*g/mL. Therefore, there can be very high serum levels of free VPA and its metabolites circulating in the blood stream that can be easily removed by hemodialysis with resultant reversal of the severe metabolic abnormalities seen in VPA toxicity [2, 4]. 

In our patient, high-flux hemodialysis was performed for four hours and found significant drop in both serum VPA and serum ammonia levels.

In conclusion, we report the successful use of high-flux hemodialysis and levocarnitine in a patient with severe acute valproic acid intoxication. The patient's hemodynamic status and mental function improved in conjunction with the acute reduction in VPA concentrations. This explanation is plausible in our case, and monitoring of phosphorus levels in patients prescribed levocarnitine may be warranted.

## Figures and Tables

**Table 1:** Arterial blood gasesses, GCS, and VPA values before and after HD.

	Before HD^*α*^	After HD^*α*^
Arterial blood gasesses		
pH	7.18	7.36
*p*O_2_ (mmHg)	75	185
*p*CO_2_ (mmHg)	48	39
HCO_3_^−^ (mmol/L)	24	22
BE (mmol/L)	−4	−3
StO_2_ (%)	80	98.3
Anion gap	25	18
Ammonia (*µ*g/dL)	325	205
GCS*	8	14
VPA (*μ*g/mL)**	928	292

*Glasgow coma scale; **valproic acid; ^*α*^hemodialysis.
